# Improvement of spontaneous locomotor activity with JAK inhibition by JTE-052 in rat adjuvant-induced arthritis

**DOI:** 10.1186/s12891-015-0802-0

**Published:** 2015-11-06

**Authors:** Atsuo Tanimoto, Yuichi Shinozaki, Keisuke Nozawa, Yukari Kimoto, Wataru Amano, Akira Matsuo, Takayuki Yamaguchi, Mutsuyoshi Matsushita

**Affiliations:** Central Pharmaceutical Research Institute, Japan Tobacco Inc., 1-1 Murasaki-cho, Takatsuki, Osaka 569-1125 Japan

**Keywords:** JAK inhibition, JTE-052, Rheumatoid arthritis, Spontaneous locomotor activity, Pain-related behavior, Motor function, Quality of life, Adjuvant-induced arthritis

## Abstract

**Background:**

Rheumatoid arthritis (RA) is a chronic inflammatory disease that leads to joint destruction, disability, and decreased quality of life (QOL). Inhibition of Janus kinase (JAK) signaling ameliorates articular inflammation and joint destruction in animal models of RA, but its effects on behaviors indicating well-being are poorly understood. In this study, we evaluated the effect of JAK inhibition on spontaneous locomotor activity in rats with adjuvant-induced arthritis, a rodent model of RA.

**Methods:**

Arthritis was induced in male Lewis rats by a single subcutaneous injection of Freund’s complete adjuvant. The novel JAK inhibitor JTE-052 was orally administered for 7 days after the onset of arthritis.

**Results:**

Induction of arthritis suppressed the spontaneous locomotor activity of the rats. Administration of JTE-052 completely improved the spontaneous locomotor activity, with partial reductions in articular inflammation and joint destruction. Hyperalgesia and motor functions were also improved, but the efficacy was not complete. However, serum interleukin (IL)-6 levels were completely decreased at 4 h after administration of the first dose of JTE-052.

**Conclusions:**

This study demonstrated that JAK inhibition improved the spontaneous locomotor activity of rats with adjuvant-induced arthritis, in association with amelioration of pain and physical dysfunction as a consequence of suppression of joint inflammation. Moreover, although further studies are needed, there was possible participation of IL-6 downregulation in the improvement of locomotor activity by JAK inhibition.

## Background

Rheumatoid arthritis (RA) is a chronic inflammatory disease of the distal joints characterized by hypertrophy and hyperplasia of the synovial epithelium, destruction of cartilage and bone, and joint infiltration by inflammatory cells such as T cells, monocytes/macrophages, and neutrophils. The quality of life (QOL) of RA patients is affected by pain and joint dysfunction. Cytokines such as interleukin (IL)-6, tumor necrosis factor (TNF)-α, IL-15, and granulocyte/macrophage-colony stimulating factor have been implicated in the pathogenesis of RA [1].

Janus kinase (JAK) is a non-receptor type tyrosine kinase family composed of four enzymes (JAK1, JAK2, JAK3, and Tyk2) that transduce signaling from multiple type I and type II cytokine receptors and mediate various inflammatory responses [2]. Several small-molecule JAK inhibitors are currently in clinical development for the treatment of transplant rejection, hematopoietic disorders, and autoimmune and inflammatory diseases, including RA [3]. Among them, tofacitinib, which was launched in 2012 for patients with severe or moderate RA, demonstrated significant efficacy for RA in multiple Phase II and Phase III trials, attenuated RA symptoms and structural damage, and improved patient QOL and physical function indexes (HAQ-DI and SF-36) [4, 5]. Preclinical studies showed that JAK inhibitors were efficacious in animal models of RA, such as adjuvant-induced arthritis (AIA) and collagen-induced arthritis (CIA), and improved articular inflammation and joint destruction [6–8]. However, the effects of JAK inhibitors on behaviors indicating well-being in animal models of RA are poorly understood.

Millecamps et al*.* [9] advocated the testing of spontaneous behaviors in rats with chronic inflammation as a new mode for global assessment of well-being in preclinical studies. Using this type of assessment, a decline in spontaneous locomotor activity was suggested to arise through pain and motor dysfunction in rat arthritis models [9–11]. Recently, we developed a novel potent JAK inhibitor, JTE-052, that was orally active in a rodent model of RA [12] and as effective as other JAK inhibitors such as tofacitinib. In the present study, we investigated the effect of JAK inhibition on spontaneous locomotor activity, and its relationships with joint inflammation and pain- and motor-related behaviors in a rat AIA model using the novel JAK inhibitor JTE-052.

## Methods

### Animals

Lewis rats were obtained from Charles River Japan (Atsugi, Japan) and maintained under specific pathogen-free conditions at a room temperature of 23 ± 3 °C and air humidity of 55 ± 15 % on a 12-h/12-h light/dark cycle. All procedures related to the use of animals in this study were reviewed and approved by the Institutional Animal Care and Use Committee of Japan Tobacco Inc.

### Compounds

JTE-052 was synthesized at the Central Pharmaceutical Research Institute, Japan Tobacco Inc. (Osaka, Japan). In an enzymatic assay, JTE-052 inhibited JAK1, JAK2, JAK3, and Tyk2 with IC_50_ values of 2.8, 2.6, 13, and 58 nM, respectively [12]. Methotrexate hydrate (MTX) was purchased from Sigma-Aldrich (St. Louis, MO). For in vivo experiments, JTE-052 and MTX were suspended in 0.5 % (w/v) methylcellulose solution.

### Induction of AIA

Arthritis was induced in the Lewis rats as previously described [13]. Briefly, heat-killed *Mycobacterium tuberculosis* H37Ra (Difco Laboratories, Detroit, MI) was suspended at 5 mg/mL in liquid paraffin, and the rats were injected with 0.1 mL of the suspension into the base of the tail on day 1 under anesthesia. The test drugs were given orally once daily from day 15 to day 21. As an index of paw swelling, the increase in hind paw volume from baseline was measured by a water displacement method using a plethysmometer for rats (Muromachi Kikai Co. Ltd., Tokyo, Japan). The rats were euthanized on day 22, and their hind paws were excised for X-ray analysis or histological evaluation. Radiographs of the right hindlimbs were obtained with a microfocal cone-beam X-ray CT scanner (MCT-CB100MF; Hitachi Medical Corporation, Tokyo, Japan). The severity of bone destruction was scored for the tarsal bone and calcaneal bone on a four-point scale from 0 to 3 (0: normal; 1: mild; 2: moderate; 3: severe). For histological analysis, the left hindlimbs were fixed in formalin, sectioned, and stained with hematoxylin and eosin. The histology of the tarsal joints was assessed using the following parameters defined in a preliminary examination: inflammatory cell infiltration; synovial cell hyperplasia; cartilage destruction; and bone destruction. The severity of each histological change was scored on a five-point scale from 0 to 4 (0: normal; 1: minimal solitary (and very small) lesions; 2: slight focal (and small) lesions; 3: moderate scattered lesions; 4: severe extensive lesions).

### Locomotor activity

The spontaneous locomotor activity of the rats was assessed using a SUPERMEX apparatus (Muromachi Kikai Co. Ltd., Tokyo, Japan) between 8:00 p.m. on day 21 and 8:00 a.m. on day 22 (dark cycle). Each rat was placed in an individual automated activity box, comprising a polycarbonate box (width × depth × height: 263 × 426 × 202 mm) placed under external sensor units. The rats were kept in the boxes for more than 1 h prior to measurement of locomotor activity to exclude typical exploratory behavior.

### Measurement of hyperalgesia

Mechanical hyperalgesia was assessed on day 22 by measuring the paw withdrawal threshold (PWT) as previously described [14] using a pressure analgesymeter (Unicom, Chiba, Japan).

### Inclined plane test and gait disturbance score

The motor function of the rats was evaluated by the inclined plane test and gait disturbance score on day 22. The inclined plane test was performed as previously described [15]. Briefly, the rats were placed on a 25° inclined plane (model SN-453; Shinano Co. Ltd., Tokyo, Japan) in the head-up position and the angle of the inclined plane was increased at a rate of 2.5°/s from 30° to 60°. The maximum angle was determined at the moment when a limb of the rat moved to maintain the body position. The test was carried out three times and the mean value was calculated. The gait disturbance of the rats was scored for each hind paw as follows: 0: no gait disturbance; 1: significant disturbance in the hind paw; 2: paralysis of the hind paw. The scores were then totaled.

### Measurement of serum IL-6

At 4 h after the initial administration of the test drugs on day 15, blood samples were collected from the tail vein. The serum IL-6 levels were quantified using an enzyme-linked immunosorbent assay kit (Quantikine ® Rat IL-6 Immunoassay kit; R&D Systems Inc., Minneapolis, MN).

### Statistical analysis

Data are expressed as means ± SD of the indicated numbers of samples. Statistical significance was assessed by Dunnett’s test (for homoscedastic data) or the Steel test (for heteroscedastic data) after homoscedasticity analysis by Bartlett’s test. For radiographic or histological scores, statistical significance was assessed by the Steel test. For correlation analyses between spontaneous locomotor activity and other parameters, statistical significance was assessed by the Spearman test.

## Results

### JAK inhibition with JTE-052 suppresses inflammation and joint destruction in AIA rats

**Fig. 1:**
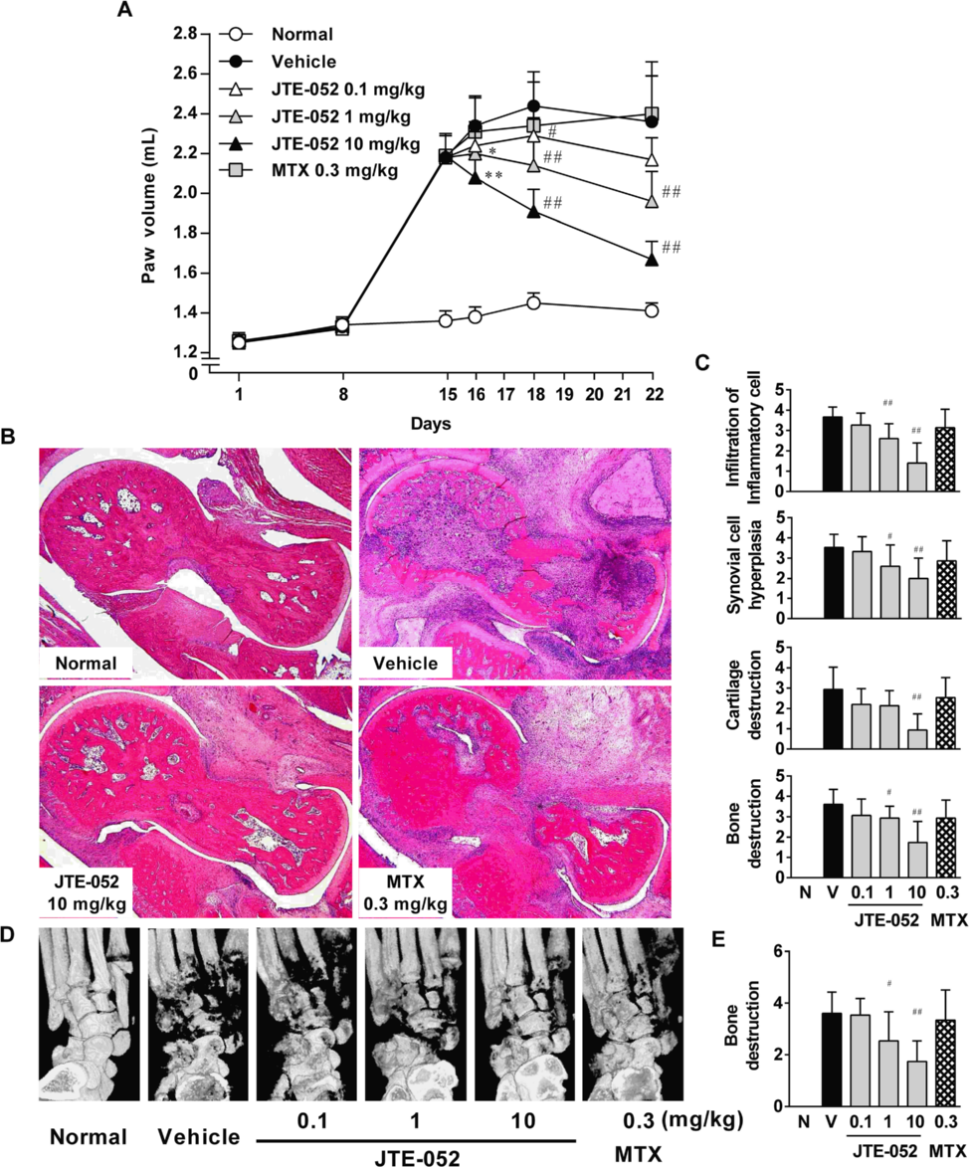
JAK inhibition with JTE-052 suppresses joint inflammation and joint destruction in rats with adjuvant-induced arthritis (AIA). Rat with clinical signs of joint inflammation were orally administered vehicle, JTE-052 (0.1, 1, or 10 mg/kg), or methotrexate hydrate (MTX; 0.3 mg/kg) once daily for 1 week from day 15. The volumes of the hind paws (**a**) were measured periodically. Hematoxylin and eosin-stained sections (**b**) and radiographs (**d**) of the hindlimbs are shown. The histological (**c**) and radiographic (**e**) changes were scored. The results are expressed as means ± SD (n = 15). *p < 0.05, **p < 0.01, versus vehicle by Dunnett’s test. ^#^ *p* < 0.05, ^##^ *p* < 0.01, versus vehicle by the Steel test

To confirm the effects of JTE-052 on inflammatory arthritis as a therapeutic treatment, we employed the rat AIA model, as a widely used animal model for RA. AIA was induced by injection of an *M. tuberculosis* suspension on day 1, and JTE-052 was orally administered once daily for 7 days from the onset of arthritis (day 15). MTX, an anchor drug for clinical treatment of RA, was used as a reference drug. As shown in Fig. 1a, JTE-052 significantly attenuated the paw swelling in a dose-dependent manner. Histological analysis of the hind paws confirmed the presence of inflammatory cell infiltration, synovial cell hyperplasia, cartilage destruction, and bone destruction in AIA rats on day 22, and revealed that JTE-052 attenuated these symptoms in a dose-dependent manner (Fig. 1b and c). Radiographic assessment of the hind paws indicated bone destruction in the tarsal and calcaneal bones of AIA rats on day 22, which was significantly attenuated by JTE-052 (Fig. 1d and e). Meanwhile, MTX at 0.3 mg/kg had no significant effects on all of these symptoms in AIA rats, despite the complete suppression of arthritis development when administered preventively in AIA rats (data not shown).

### JAK inhibition with JTE-052 improves spontaneous locomotor activity in AIA rats

**Fig. 2:**
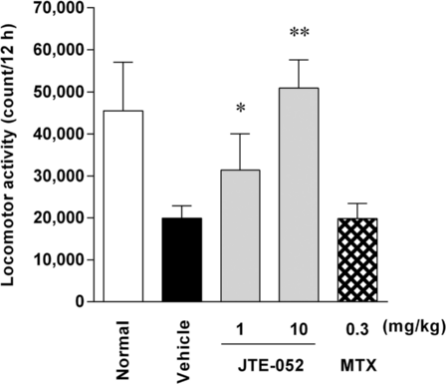
JAK inhibition with JTE-052 improves spontaneous locomotor activity in rats with adjuvant-induced arthritis (AIA). The spontaneous locomotor activity of AIA rats was assessed using a SUPERMEX apparatus between 8:00 p.m. on day 21 and 8:00 a.m. on day 22. The results are expressed as means ± SD (n = 5). *p < 0.05, **p < 0.01, versus vehicle by Dunnett’s test

Next, we examined the effect of JTE-052 on spontaneous locomotor activity. Induction of AIA significantly decreased the spontaneous locomotor activity from day 15 compared with non-AIA rats (16855 ± 5021 vs. 46897 ± 11215 counts/12 h), and the decrease was observed until day 22 (19890 ± 2953 vs. 45508 ± 11525 counts/12 h). JTE-052 dose-dependently ameliorated the spontaneous locomotor activity, and complete remission was observed at a dose of 10 mg/kg on day 22 when administered after the onset of arthritis (Fig. 2). MTX had no significant effect on the spontaneous locomotor activity when administered after the onset of arthritis. The ameliorating effect of JTE-052 on the spontaneous locomotor activity was correlated with that on paw swelling, as revealed by a correlation analysis between paw volume and spontaneous locomotor activity (*r*^2^ = 0.85, *p* < 0.01).

### JAK inhibition with JTE-052 ameliorates the hyperalgesia in AIA rats

**Fig. 3:**
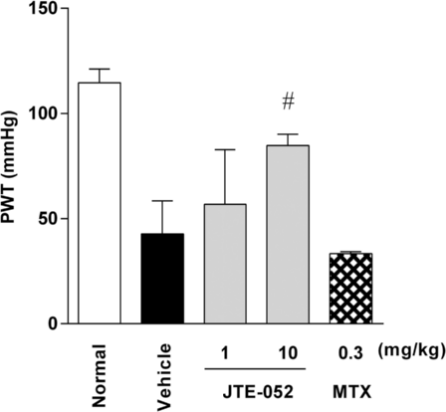
JAK inhibition with JTE-052 ameliorates hyperalgesia in rats with adjuvant-induced arthritis (AIA). The mechanical hyperalgesia of AIA rats was assessed by measuring the paw withdrawal threshold (PWT) on day 22. The results are expressed as means ± SD (*n* = 5). ^#^ *p* < 0.01, versus vehicle by the Steel test

To investigate the underlying mechanism by which JTE-052 ameliorates the decrease in spontaneous locomotor activity in AIA rats, the effect of JTE-052 on the mechanical hyperalgesia of AIA rats when administered after the onset of arthritis was evaluated. Induction of AIA significantly decreased the PWT of the hind paw from day 14 compared with non-AIA rats (39.1 ± 6.7 vs. 111.0 ± 21.0 mmHg), and the decrease was observed until day 22 (42.7 ± 15.8 vs. 114.6 ± 6.6 mmHg). JTE-052 ameliorated the decrease in PWT in a dose-dependent manner (Fig. 3), but the efficacy was not complete. MTX had no significant effect on the hyperalgesia. The ameliorating effect of JTE-052 on the hyperalgesia was correlated with that on spontaneous locomotor activity, as revealed by a correlation analysis between PWT and spontaneous locomotor activity (*r*^2^ = 0.77, *p* < 0.01).

### JAK inhibition with JTE-052 ameliorates the motor function in AIA rats

**Fig. 4:**
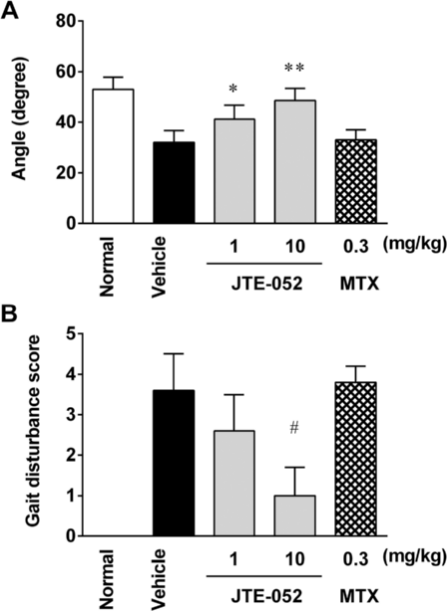
JAK inhibition with JTE-052 ameliorates motor function in rats with adjuvant-induced arthritis (AIA). An inclined plane test (**a**) was performed and gait disturbance (**b**) was scored in rats on day 22. The results are expressed as means ± SD (*n* = 5). *p < 0.05, **p < 0.01, versus vehicle by Dunnett’s test. ^#^ *p* < 0.01, versus vehicle by the Steel test

To further investigate the underlying mechanism by which JTE-052 ameliorates the decrease in spontaneous locomotor activity in AIA rats, the effect of JTE-052 on the motor function of AIA rats when administered after the onset of arthritis was investigated. The inclined plane test was performed as an index of motor function. The maximum angle for AIA rats decreased from day 15 compared with non-AIA rats (28.3 ± 2.6° vs. 49.6 ± 4.6°), and the decrease was observed until day 22 (32.0 ± 4.7° vs. 53.0 ± 4.8°). JTE-052 ameliorated the decrease in the maximum angle on day 22 in a dose-dependent manner (Fig. 4a). Next, the gait disturbance was investigated as a separate index of motor function. The gait disturbance score was increased in AIA rats from day 15 compared with non-AIA rats (3.2 ± 0.8 vs. 0.0 ± 0.0), and continued to increase until day 22 (3.6 ± 0.9 vs. 0.0 ± 0.0). JTE-052 decreased the gait disturbance score in a dose-dependent manner (Fig. 4b). MTX had no significant effect on the maximum angle in the inclined plane test or the gait disturbance score (Fig. 4a and b). The ameliorating effects of JTE-052 on both the inclined plane test and the gait disturbance score were correlated with that on spontaneous locomotor activity, as revealed by correlation analyses between the maximum angle or gait disturbance score and spontaneous locomotor activity (*r*^2^ = 0.61, *p* < 0.01 and *r*^2^ = 0.81, *p* < 0.01, respectively).

### JTE-052 decreases serum IL-6 levels

**Fig. 5:**
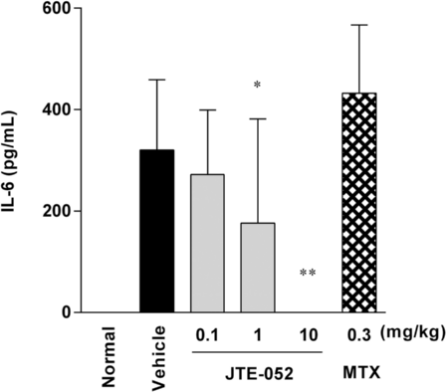
JAK inhibition with JTE-052 decreases the serum IL-6 levels in rats with adjuvant-induced arthritis (AIA). The serum IL-6 levels were assessed at 4 h after the first administration of JTE-052 on day 15. The results are expressed as means ± SD (*n* = 15). *p < 0.05, **p < 0.01, versus vehicle by Dunnett’s test

To investigate the effect of JTE-052 on a fatigue-related inflammatory mediator, the effect of JTE-052 on the serum IL-6 levels of AIA rats when administered after the onset of arthritis was analyzed. The serum IL-6 levels were increased in AIA rats compared with non-treated rats, and JTE-052 suppressed the serum IL-6 levels at 4 h after the initial dose on day 15 (Fig. 5).

## Discussion

In this study, we investigated the effect of the novel JAK inhibitor JTE-052 on arthritis-induced behavioral changes in an animal model of RA. Oral administration of JTE-052 to AIA rats improved their spontaneous locomotor activity, which was correlated with the amelioration of articular inflammation, hyperalgesia, and motor function. In addition, a decrease in serum IL-6 was observed after the first administration of JTE-052.

JAK inhibition by JTE-052 inhibited joint inflammation and joint destruction when administered after the onset of arthritis. These findings are consistent with previous reports on two other JAK inhibitors, tofacitinib and baricitinib [7, 8, 16]. Although JAK inhibition with all three JAK inhibitors showed rapid and strong suppressive effects on articular inflammation and joint destruction when administered around the peak of arthritis, the effects on the symptoms were not complete (Fig. 1a) [7, 8]. Dosing with JTE-052 at 10 mg/kg from the day before the induction of arthritis completely inhibited AIA (data not shown) or CIA [12] in rats. Therefore, it was thought that a dose of 10 mg/kg JTE-052 would be sufficient to completely inhibit cytokine signaling. A previous study reported that mediators other than JAK-related cytokines, such as TNF-α, might participate in the pathogenesis of rat AIA [17]. Serum TNF-α was also reported to be increased after the onset of arthritis in AIA rats [18]. These observations might explain the incomplete efficacy of JAK inhibitors on AIA in rats when administered after the onset of arthritis. Under the same conditions, MTX failed to show inhibitory effects at a dose that produced a complete inhibitory effect when administered before the onset of arthritis (data not shown). Currently, several pharmacological mechanisms of action for MTX have been proposed, and reduction of antigen-dependent T cell proliferation is largely responsible for its anti-rheumatic effect [19]. D-penicillamine and auranofin were also reported to inhibit T cell proliferation [20, 21], but had no inhibitory effects on a rat AIA model when administered after the onset of arthritis [22]. These findings indicate that anti-arthritic drugs that inhibit T cell proliferation do not ameliorate AIA when administered after disease onset. However, JAK inhibitors were reported to inhibit the activation of many types of inflammatory cells, including B cells, neutrophils, and macrophages, as well as that of T cells [12, 23, 24], indicating that JAK inhibition by JAK inhibitors is even efficacious when the inhibitors are administered after the onset of arthritis.

In the present study, JAK inhibition by JTE-052 ameliorated the spontaneous locomotor activity in AIA rats, and complete improvement was seen at a dose of 10 mg/kg. A decline in spontaneous locomotor activity was observed concurrently with reduced paw swelling in AIA rats, suggesting that inhibition of articular inflammation by JAK inhibition was a major cause of the improvement in spontaneous locomotor activity. Nevertheless, JTE-052 completely improved the spontaneous locomotor activity despite its partial inhibition of articular inflammation. It has been reported that tacrolimus improved spontaneous locomotor activity without amelioration of paw swelling when administered after the onset of arthritis in a rat CIA model [10]. Thus, factors other than articular inflammation may also participate in the improvement of locomotor activity by JAK inhibition. Therefore, we investigated the effects of JAK inhibition on factors that appeared to contribute to the reduced locomotor activity, such as pain, physical function, and fatigue, as important factors for QOL in RA patients.

Pain is the most important problem in patients with RA, and reduces their QOL [25, 26]. Administration of analgesic agents was reported to improve the spontaneous locomotor activity in AIA rats [11], suggesting that inhibition of hyperalgesia by JTE-052 is a likely cause of the improvement in spontaneous locomotor activity. However, the amelioration of hyperalgesia by JTE-052 was not complete, similar to the case for articular inflammation. Although several pain-producing substances, such as bradykinin, nerve growth factor, prostaglandins, and ATP, were reported to be involved in inflammatory pain [27], JAK provides little contribution to the signaling of these substances. Therefore, it is suggested that the inhibition of hyperalgesia by JAK inhibition arises through inhibition of inflammation, and that JAK inhibition does not ameliorate spontaneous locomotor activity through direct inhibition of hyperalgesia in AIA rats.

It has been reported that physical dysfunction can arise through joint destruction resulting from cartilage and bone destruction, and decrease the QOL of RA patients [28, 29]. Although there are few available reports, it is easy to imagine the correlation between physical function and spontaneous locomotor activity. Therefore, in this study, we evaluated the effect of JAK inhibition on motor function by the inclined plane test and gait disturbance score to investigate the physical dysfunction. Although JAK inhibition by JTE-052 ameliorated the outcomes of both tests, the amelioration of motor function was not complete and was comparable to the amelioration of cartilage and bone destruction. In addition, the amelioration of joint destruction by JTE-052 was comparable to the amelioration of joint inflammation, suggesting that JAK inhibition by JTE-052 ameliorated joint destruction through the inhibition of joint inflammation, rather than having a direct inhibitory effect on joint destruction. These findings are consistent with a previous report describing that JAK inhibition by tofacitinib ameliorated joint destruction by inhibiting the activation of inflammatory cells expressing RANKL and had no impact on osteoclast differentiation and function [8]. Taken together, these findings indicate that inhibition of motor function by JAK inhibition arises as a result of suppression of joint destruction through the inhibition of joint inflammation, and that JAK inhibition does not ameliorate spontaneous locomotor activity through direct inhibition of physical function in AIA rats.

Fatigue is commonly reported in RA patients, with 41 % experiencing clinically important levels of fatigue [30], and can affect their QOL. In patients with RA, TNF-α and IL-1 were thought to be the primary cytokines contributing to the pathogenesis [31], and biologics targeting these cytokines were reported to reduce fatigue [32, 33]. However, systemic upregulation of TNF-α was not as high and upregulation of IL-1 was not observed in AIA rats [18]. In contrast, other pro-inflammatory cytokines such as IL-6, IL-17, transforming growth factor-β, and IL-18 were clearly upregulated in AIA rats [18] and considered to contribute to the pathogenesis of joint inflammation [34, 35]. Among them, IL-6 is another cytokine that has been suggested to induce fatigue in RA patients [36]. Furthermore, a tumor-induced decrease in spontaneous locomotor activity was correlated with the systemic levels of IL-6 in mice [37]. As JAK is associated with the IL-6 receptor and has a role in signaling [38], inhibition of JAK is thought to block IL-6 signaling. In AIA rats, production of IL-6 was observed in association with articular inflammation and was reduced by JTE-052 (Fig. 5). Therefore, a direct reduction of fatigue via inhibition of IL-6 production as well as blockade of IL-6 signaling by JAK inhibition may be involved in the improvement of spontaneous locomotor activity in AIA rats. Moreover, these findings suggest that the clinical improvement in QOL of RA patients by JAK inhibitors such as tofacitinib might include the improvement of fatigue by inhibition of IL-6. Because the reduced spontaneous locomotor activity may involve not only pain and physical function induced by joint inflammation and joint destruction, but also fatigue induced by systemic inflammation, which are components of decreased QOL in humans, measurement of spontaneous locomotor activity in animal models of RA might be a useful index to assess the ameliorating actions of agents on the QOL of RA patients.

## Conclusions

In conclusion, we have demonstrated that JAK inhibition by JTE-052 improved spontaneous locomotor activity in AIA rats. The improvement was accompanied by amelioration of pain and physical dysfunction as a consequence of suppression of articular inflammation and joint destruction. Moreover, although further studies are needed, there was possible participation of IL-6 downregulation in the improvement of spontaneous locomotor activity by JAK inhibition.

## Abbreviations

AIA: adjuvant-induced arthritis
CIA: collagen-induced arthritis
IL: interleukin
JAK: Janus kinase
MTX: methotrexate hydrate
PWT: paw withdrawal threshold
QOL: quality of life
RA: rheumatoid arthritis
TNF: tumor necrosis factor
